# The Development and Study of Recombinant Immunoglobulin A to Hemagglutinins of the Influenza Virus

**Published:** 2018

**Authors:** T. K. Aliev, I. G. Dement’yeva, V. A. Toporova, V. V. Argentova, L. P. Pozdnyakova, M. N. Bokov, Yu. A. Votchitseva, D. A. Dolgikh, S. D. Varfolomeyev, P. G. Sveshnikov, M. P. Kirpichnikov

**Affiliations:** Lomonosov Moscow State University, Department of Chemistry, Leninskie gory 1, bldg. 3, Moscow, 119991, Russia; Russian Research Center for Molecular Diagnostics and Therapy, Simferopolsky Blvd. 8, Moscow, 117149 , Russia; Shemyakin-Ovchinnikov Institute of Bioorganic Chemistry, Russian Academy of Sciences, Miklukho-Maklaya Str. 16/10, Moscow, 117997, Russia; Lomonosov Moscow State University, Faculty of Biology, Leninskie gory 1, bldg. 12, Moscow, 119991 , Russia

**Keywords:** Influenza A virus, broadly neutralizing antibodies, immunoglobulin A, IgA1, IgA2m1, recombinant antibodies

## Abstract

We obtained recombinant variants of human antibody FI6 broadly specific to hemagglutinins of the influenza A virus. On the basis of a bi-promoter (CMV, hEF1-HTLV) vector, we developed genetic constructs for the expression of the heavy and light chains of the immunoglobulins of IgA1-, IgA2m1-, and IgG-isotypes. Following transfection and selection, stable Chinese hamster ovary (CHO) cell lines were produced. The antibodies of IgA1-, IgA2m1-, and IgG-isotypes were purified from culture media. We performed an immunochemical characterization and studied their interactions with influenza A strains of the H1N1- and H3N2-subtypes. It was shown that recombinant FI6 variants of the IgA-isotype retain the properties of the parental IgG antibody to demonstrate specificity to all the strains tested. The strongest binding was observed for the H1N1 subtype, which belongs to hemagglutinins of phylogenetic group I.

## INTRODUCTION

Passive immunotherapy with antibodies targeting viral capsid components is a promising strategy in the design of new drugs against influenza viruses [1, 2]. This approach is of particular importance because of the high antigenic variation of surface influenza A virus (IAV) proteins that decreases the efficacy of vaccines and low-molecular therapeutic agents. In recent years, neutralization of monoclonal antibodies (mABs) cross-protective against diverse IAV serotypes have been pursued in the design of broad spectrum antivirals [3-7]. A large-scale screening of more than 100,000 individual, cultured antibody-producing B cells selected from several donors with significant heterosubtypic immunity against several IAV subtypes, has been a great success [8]. A unique antibody FI6 that targets the recombinant and natural hemagglutinins of phylogenetic groups I and II was found. The broad specificity of this antibody appears to be associated with the targeting of a conserved epitope in the F subdomain of hemagglutinin, which is less mutation-prone than the HA1 domain. Transfer of this antibody at a dose of 2–20 mg/kg into mice and ferrets after lethal H1N1 and H5N1 challenge conferred full protection. The discovery of this broad-spectrum antibody opens up myriad opportunities for the creation of different recombinant immunoglobulins on its basis.

The respiratory tract is the major route for IAV entry into host cells; hence, intranasal administration of neutralizing antibodies can significantly enhance the effect of passive immunotherapy [9, 10]. The intranasal delivery of recombinant immunoglobulin A, the most prevalent antibody at human mucosal sites, appears as a very intriguing option [11]. Class A immunoglobulins come in various isoforms (monomer, dimer, secretory form) and, thus, employ different mechanisms for virus neutralization. IgA-isotype antibodies can block virus interaction with the surface of human cells, neutralize the viral particles inside cells, and facilitate the destruction of infected cells by attracting and activating neutrophils [12].

The aim of this study was to produce recombinant variants of antibody FI6 of IgA-isotype and to compare its immunochemical properties with those of IgG-isotype.

## EXPERIMENTAL

**Construction of the bi-promoter vector for the expression of recombinant FI6 IgG1-isotype antibodies**

We had previously synthesized [13] cDNA sequences of the variable domain of the heavy FI6VHv3 and light FI6VKv2 chains of antibody FI6 [8].

A fragment with a nucleotide sequence encoding the leader peptide MAWVWTLLFLMAAAQSAQA and the untranslated regulatory region were fused to the 5’-end region of the previously synthesized cDNA of the heavy chain variable domain FI6VHv3 through splicing by overlap extension polymerase chain reaction (SOE-PCR). To create the bi-promoter expression cassette, the SOE-PCR DNA fragment was treated with NheI and Bsp120I restrictases and cloned into the pSK+/hEF1-HTLV-BGH plasmid, pretreated with the same restrictases [14] containing the hEF1-HTLV hybrid promoter, the full-length IgG1 constant region and polyadenylation site BGH, all flanked with MluI restriction sites. Thus, a pSK+/hEF1-HTLV-FI6HG1- BGH plasmid was obtained.

Similar to the heavy chain, human antibody light chain cDNA adapted to expression in eukaryotic cells was obtained. Using SOE-PCR, we spliced cDNA of the leader peptide MKSQTQVFVFLLLCVSGAHG, previously synthesized cDNA of the variable domain of the FI6VKv2 light chain, and cDNA of the constant domain of human kappa-isotype. The resulting DNA fragment was treated with NheI and Sfr274I restrictases and cloned into the pOptiVEC plasmid (Invitrogen, USA) that was pretreated with the same restrictases and carried a preliminarily inserted MluI restriction site near the 5’-end of the promoter. In this way, a pOpti-FI6L plasmid containing the light-chain gene of antibody FI6 under the cytomegalovirus promoter (CMV) control was obtained.

At the final stage of pBiPr-ABIgG1FI6 bi-promotor plasmid creation, the MluI-MluI fragment (2500 bp) from the pSK+/hEF1-HTLV-FI6HG1-BGH plasmid was inserted into the pOpti-FI6L dephosphorylated vector pretreated with the MluI restrictase.

**Construction of bi-promoter plasmids for the expression of recombinant FI6 IgA1- and IgA2m1-isotype antibodies**

The constant heavy-chain domains of IgA1- and IgA2m1-isotopes were obtained as follows. Exons of the corresponding genes were amplified using the human chromosomal DNA template and specific oligonucleotide primers and cloned into an intermediate vector, pAL-TA (Eurogen, Russia). Exons of the constant domains of the same isotopes were spliced by SOE-PCR. The obtained fragments were treated with SacI and Sfr274I restrictases; each of these fragments, the NheI-SacI fragment from pSK+/hEF1- HTLV-FI6HG1-BGH containing cDNA of the leader peptide MAWVWTLLFLMAAAQSAQA and cDNA of the variable heavy-chain region of antibody FI6, was cloned into the pSK+/hEF1-HTLV-BGH vector pretreated with NheI and Sfr274I restrictases. Thus, the plasmids pSK+/hEF1-HTLV-FI6HA1-BGH and pSK+/hEF1-HTLV-FI6HA2m1-BGH were obtained that contained the hEF1-HTLV promoter, cDNA of the leader peptide MAWVWTLLFLMAAAQSAQA, the variable heavy-chain region of antibody FI6, cDNA of the constant domain of human IgA1- or IgA2m1-isotype (respectively), and an untranslated region which includes the polyadenylation site BGH flanked by MluI restriction sites.

At the final stage of creation of pBiPr-ABIgA1FI6 and pBiPr-ABIgA2m1FI6 bi-promotor vectors, fragments MluI-MluI (2500 bp) from the plasmids pSK+/hEF1-HTLV-FI6HA1-BGH and pSK+/ hEF1-HTLV-FI6HA2m1-BGH, respectively, were inserted into the pOpti-FI6L dephosphorylated vector pretreated with MluI restrictase.

**Preparation of cell lines producing recombinant antibodies**

CHO DG44 cells (Invitrogen, USA) were transfected with linearized pBiPr-ABIgG1FI6, pBiPr-ABIgA1FI6, and pBiPr-ABIgA2m1FI6 plasmids using the Lipofectamine 3000 reagent (Invitrogen, USA) according to the standard protocol. Primary selection of transfected cells was performed using the CD OptiCHO medium (Invitrogen, USA) with addition of 8 mM *L*-glutamine (Gibco, USA), 0.1% Pluronic F-68 (Gibco, USA), and a 1X antibiotic/antimycotic solution (Gibco, USA). Fluorescent screening and selection of the producer clones were performed to obtain a stable cell line. The cells were seeded on a semi-solid CloneMedia medium (Molecular Devices, USA) with the addition of mouse antibodies to the constant domains of human immunoglobulins G (Molecular Devices, USA) or A (Russian Research Center for Molecular Diagnostics and Therapy, Russia) depending on the isotype of the FITC-labeled recombinant antibodies. After 14 days of cell cultivation, some producing clones were selected using the ClonePix FL device (Molecular Devices, USA) based on fluorescence intensity. The selected clones were cultured in the presence of increasing methotrexate concentrations from 20 to 500 nM to enhance productivity.

**Extraction and purification of recombinant antibodies**

The culture of cells producing recombinant antibodies was grown in spinner flasks with a 500 mL working volume. For this, 2.5–3.0 × 10^5^ cells/mL were seeded in a 300 mL CD OptiCHO medium and grown for 14– 18 days in a CO_2_-incubator at 37°C, 8% CO_2_ and a stirring rate of the spinner of 50–70 rpm.

The culture fluid was centrifuged at 4000 *g; *50 mM 2-(N-morpholino)ethanosulfonic acid (MES) and 150 mM NaCl were added to the supernatant, pH 5.7.

The culture fluid containing FI6-IgG was loaded on the Protein G-Sepharose 4B Fast Flow column (diameter 2.5 cm, gel height 3.5 cm, volume 17 mL), pre-equilibrated with a MES solution pH 5.7, at a recirculation rate of 42 mL/h (8.6 mL/h × cm^2^) for 21 h at 4°C. Antibodies were eluted with 0.1 M glycine buffer at pH 2.7 and an elution rate of 70 mL/h. Immediately after the eluate was obtained, pH was adjusted to ~7.5 with 2 M Tris and concentrated using the 30000 NMWL ultrafiltration membrane to a volume of ≈1.5–2 mL and dialyzed against a phosphate buffer (200X volume) at pH 7.4 overnight.

For affine chromatography of FI6-IgA1 and FI6-IgA2m1, an immunosorbent based on FabH A3 mouse monoclonal antibodies (mABs) (Russian Research Center for Molecular Diagnostics and Therapy, Russia) to human immunoglobulin kappa-chain was obtained. Antibodies were immobilized on activated BrCN-sepharose according to Kavran et al. [15]. The immobilization degree of FabH A3 antibodies was 5 mg per 1 mL of sepharose. The pH of the culture fluid containing FI6-IgA antibodies was adjusted to 8.0 with a 1 M Tris solution and loaded on the column via recirculation for 18 h at a rate of 15 mL/h. For the elution of the FI6-IgA1 and FI6-IgA2m1 antibodies, 0.1 M sodium-acetate buffer, pH 3.0; 0.5 M NaCl; 0.1 M glycine buffer, pH 2.5; 0.5 M NaCl; 0.1 M glycine buffer, pH 2.0; 0.5 M NaCl were consecutively used. All eluates were neutralized with a 1 M Tris solution.

**Immunochemical analysis of recombinant antibodies**

In this work, we used highly purified relic and current strains of IAV produced by Hytest Ltd. (Turku, Finland) and the Research Institute of Influenza RAMS (St. Petersburg, Russia) obtained from infected chicken embryos by successive ultracentrifugation in a sucrose density gradient and inactivation with merthiolate for 24 hours (*Table 1*). Virus inactivation was confirmed on a MDCK cell culture.

**Table 1:** Characterization of the viral samples used in the work

Manufacturer	Serotype	Strain/year of isolation
Hytest Ltd 8IN73	Influenza A (H1N1)	A/Taiwan/1/86
Hytest Ltd 8IN73-2	Influenza A (H1N1)	A/Beijing/262/95
Hytest Ltd 8IN73-3	Influenza A (H1N1)	A/New Caledonia/20/99
Hytest Ltd 8IN73-4	Influenza A (H1N1)	A/Solomon Islands/03/06
Research Institute of Influenza	Influenza A (H1N1)	A/California/07/09
Hytest Ltd 8IN74	Influenza A (H3N2)	A/Samara/222/99=A/Shangdong/9/93
Hytest Ltd 8IN74-1	Influenza A (H3N2)	A/Panama/2007/99
Hytest Ltd 8IN74-2	Influenza A (H3N2)	A/Kiev/301/94
Hytest Ltd 8IN74-3	Influenza A (H3N2)	A/Wisconsin/67/05
Hytest Ltd 8IN74-4	Influenza A (H3N2)	A/Brisbane/10/07
Research Institute of Influenza	Influenza A (H3N2)	A/Sydney/5/97
Hytest Ltd 8IN75-2	Influenza B	B/Tokio/53/99

Recombinant antibody titration was performed by indirect, enhanced light immunosorbent assay (ELISA). The inactivated IAV strains were sorbed at a concentration of 5 μg/mL at 4°C overnight in 50 μL of a 0.1 M carbonate buffer at pH 9.2–9.4 in the wells of a 96-wellplate with high binding capacity (Corning-Costar, Netherlands). FabH A3 mABs conjugated to horseradish peroxidase were used as the secondary antibody for detection.

For Western blot, electrophoretic separation of influenza A virus strain A/Solomon Islands/03/06 in 10% polyacrylamide gel upon non-reducing conditions was performed. Electrophoretic transfer (electroblotting) of proteins from the gel to the nitrocellulose membrane S045A330R (Advantec MFS, Inc., USA) was conducted. Transferred proteins were detected on the nitrocellulose membrane by indirect ELISA. The membrane was blocked with a 5% casein solution for 1 h at room temperature on a shaker, rinsed three times with PBS-T (10 mM K_2_HPO_4_, pH 7.5, 0.145 M NaCl, 0.1% Tween 20), and incubated for 1 h on a shaker at room temperature. After three times rinsing, the membrane was incubated with a solution of corresponding recombinant antibodies at a concentration of 1 μg/mL in a phosphate-salt buffer for 1 h at 37oC. After three times rinsing with PBS-T, the membrane was incubated with FabH A3 mABs conjugated to horseradish peroxidase for 1 h at 37oC. Western blots were stained by adding a substrate (3,3-diaminobenzidine, 4-chlorine-1-naphthol and hydrogen peroxide).

*K*_d_ of the antigen–antibody complex was estimated according to Friguet et al. [16]. At the first stage, mABs at a constant concentration of 1 nM (150 ng/mL) were incubated with an inactivated antigen of the influenza A(H1N1)/Solomon Islands/03/06 strain in a concentration range of 0.1–10 nM (10–1000 ng/mL) for 2 h at room temperature with constant stirring on a shaker to achieve a thermodynamic equilibrium in a three-component system: free antigen, free antibody, and a antigen–antibody complex. At the second stage, the concentrations of free antibodies were measured by solid-phase ELISA with an antigen immobilized on the wellplate. At the final stage, the *K*_d_ value was estimated using the Klotz equation [17] from the values of the total antigen concentration and free recombinant antibody concentration.

## RESULTS AND DISCUSSION

Recombinant immunoglobulins were generated using nucleotide sequences encoding the variable domains of the heavy FI6VHv3 and light FI6VKv2 chains of a broad-spectrum neutralizing antibody FI6 [8]. Such modified sequences differ from the sequences encoding the heavy and light chains of immunoglobulin FI6 by the fact that they contain less somatic mutations and correspond more to the variable domain germ-line sequences of human immunoglobulin.

Recombinant IgA1- and IgA2m1-isotype antibodies were obtained to study the ability of the FI6 antibody to interact with IAV of IgA-isotype. The IgG1-isotype antibody FI6 was obtained as a positive control.

Human immunoglobulin A comes in two isotypes – IgA1 and IgA2. IgA1 dominates in the serum, while the proportion of IgA2 is higher in secretions [18]. The most significant structural differences between these isotypes are associated with the hinge region. IgA1 has a 13-amino-acid-longer hinge than IgA2, resulting in more flexible antigen-binding sites for the IgA1-isotype antibodies. This advantage renders IgA1 more susceptible to proteolytic cleavage at the hinge region compared to IgA2 [12]. IgA2-isotype antibody comes in two allotypes: IgA2m1 and IgA2m2, which differ in the number of glycosylation sites and, most significantly, the location of inter-chain disulfide bonds [19, 20]. IgA2m1 lacks disulfide bonds between the constant domain of the light chain and the constant domain of the heavy chain (CH1), which are typical for the structure of immunoglobulins. In this case, the disulfide bond forms between the constant domains of light chains and the interaction between the light and heavy chain is non-covalent.

**Fig. 1:**
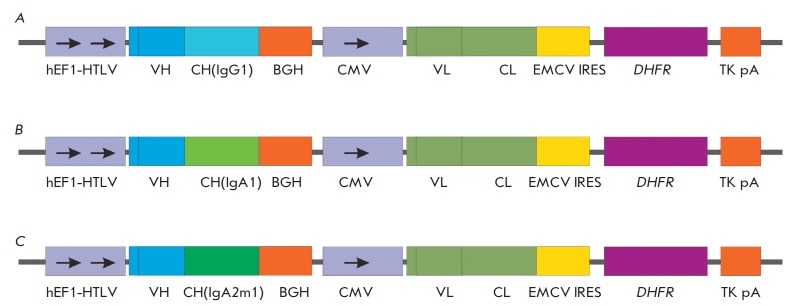
Expression cassettes of bi-promotor plasmids for the production of FI6 antibodies of different isotypes. A. Plasmid pBiPr-ABIgG1FI6 B. Plasmid pBiPr-ABIgA1FI6. C. Plasmid pBiPr-ABIgA2m1FI6. hEF1-HTLV – hybrid promotor from plasmid pMG, combining the promotor of an elongation factor EF-1a and 5’-untranslated region of the human T-cell leukemia virus HTLV; VH – variable domain of an antibody heavy chain; CH(IgG1), CH(IgA1), CH(IgA2m1) – constant domains of human immunoglobulin heavy chains of IgG1-, IgA1-, IgA2m1-isotypes, respectively; BGH – polyadenylation site BGH; CMV –promotor/enhancer of early genes of human cytomegalovirus; VL – variable domain of an antibody light chain; CL – constant domain of an antibody light chain; EMCV IRES – internal ribosome entry site (IRES) of the encephalomyocarditis virus; *DHFR *– dihydropholate reductase gene; TK pA – herpes virus thymidine kinase polyadenylation signal.

For the expression of recombinant antibodies in CHO cells, we had previously developed a bi-promoter vector which was effective in producing antibodies. This expression vector contains two transcription units, and pCMV and hEF1-HTLV promoters that control the transcription of heavy and light antibody chains in one plasmid. The plasmid also contains the dihydropholate reductase gene (*DHFR*), which is translated with an independent ribosomal binding site. During amplification of the *DHFR *gene copies in the chromosome of producer lines by means of methotrexate (MTX) selective pressure, this vector allows simultaneous increase in the light- and heavy-chain gene copy numbers. Three expression plasmids different in the constant domains of immunoglobulin heavy chains were obtained (*Fig. 1*).

**Fig. 2:**
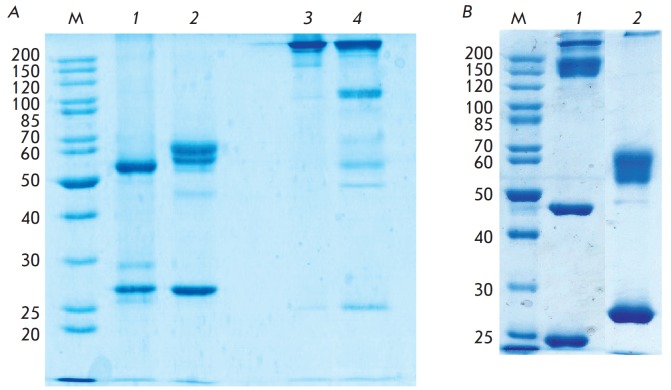
Gel electrophoresis of FI6 antibodies upon reducing and non-reducing conditions. A. 1, 3 – IgG antibodies; 2, 4 – IgA1 antibodies. 1, 2 – in the presence of β-mercaptoethanol. 3, 4 – in the absence of β-mercaptoethanol. B. IgA2m1 antibodies in the absence (1) and in the presence (2) of β-mercaptoethanol. M – molecular weight markers, kDa.

Stable cell lines based on CHO DG44 cells were generated for the production of recombinant immunoglobulins. Recombinant IgG and IgA antibodies were isolated from a serum-free culture medium. After affinity chromatography, recombinant IgG and IgA-isotype antibodies were analyzed using polyacrylamide gel electrophoresis upon reducing and non-reducing conditions (*Fig. 2*).

An analysis of gel electrophoresis showed that the size of the detected protein fragments reflects the features of the location of inter-chain disulfide bonds in each of the studied isotopes. Thus, two bands corresponding to the light and heavy chains of the immunoglobulins appear on the electrophoregrams of IgG- and IgA1-isotype antibodies upon reducing conditions. The IgA2m1-isotype antibody was found to have a unique location of the inter-chain disulfide bonds characteristic of this isotype. As mentioned previously, IgA2m1- isotype antibodies lack an inter-chain disulfide bond between the constant domain of the light chain and the CH1-domain of the heavy chain, which is common to most immunoglobulins. Herewith, the constant domains of the light chains are interconnected by a disulfide bond. Dimers of the light (~46 kDa) and heavy chains (~105 kDa) are found on gel electrophoregrams (*Fig. 2B*) upon non-reducing conditions.

**Fig. 3:**
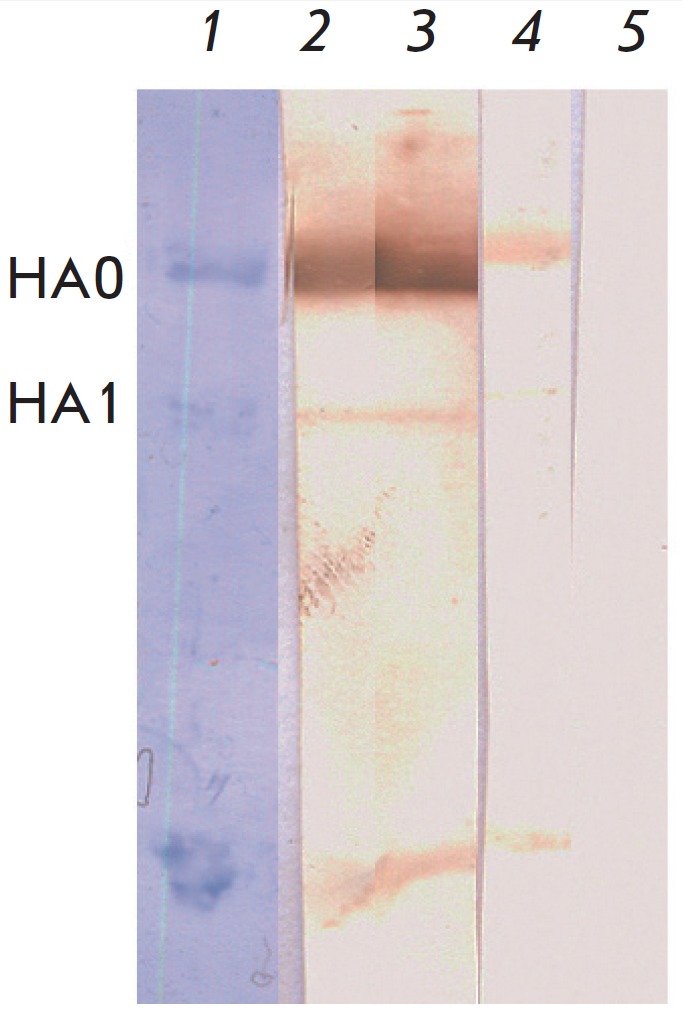
Western blot of FI6 recombinant antibodies with the proteins of the influenza A virus, strain A/Solomon Islands/03/06 (H1N1). Lane 1. Influenza A virus proteins before transfer to a membrane. Lane 2. Western blot with FI6 IgG1 antibodies. Lane 3. Western blot with FI6 IgA1 antibodies. Lane 4. Western blot with FI6 IgA2m1 antibodies. Lane 5. Conjugate control (Western blot in the absence of recombinant antibodies).

The antigen-binding activity of the recombinant proteins was studied by Western blotting with an inactivated A/Solomon Islands/03/06 H1N1 influenza strain (*Fig. 3*).

Western blot data confirm the ability of the recombinant antibodies to recognize the native hemagglutinin of IAV. Western blotting confirmed previous results on the Fab-fragment of the FI6 IgG1-isotype antibody [13] indicating that antibody FI6 can interact with both whole HA0 hemagglutinin and the HA1 and HA2 fragments formed during hydrolysis of the whole protein in gel electrophoresis upon reducing conditions [21]. These results are consistent with the data of FI6 antibody epitope mapping presented in [8]. The broad specificity of FI6 antibody occurs because that FI6 targets a conserved epitope in the F-subdomain of hemagglutinin located between the H1 and H2 domains. Herein, the heavy chain of the antibody interacts with the H1 domain and the light chain – with the alpha helix from the H2 domain.

The ability of IgA-isotype antibodies to interact with different IAV subtypes was of interest. IgA1- and IgA2m1-isotype antibodies were compared by indirect ELISA using various inactivated H1N1 and H3N2 subtype influenza A strains immobilized in solid phase.

**Fig. 4:**
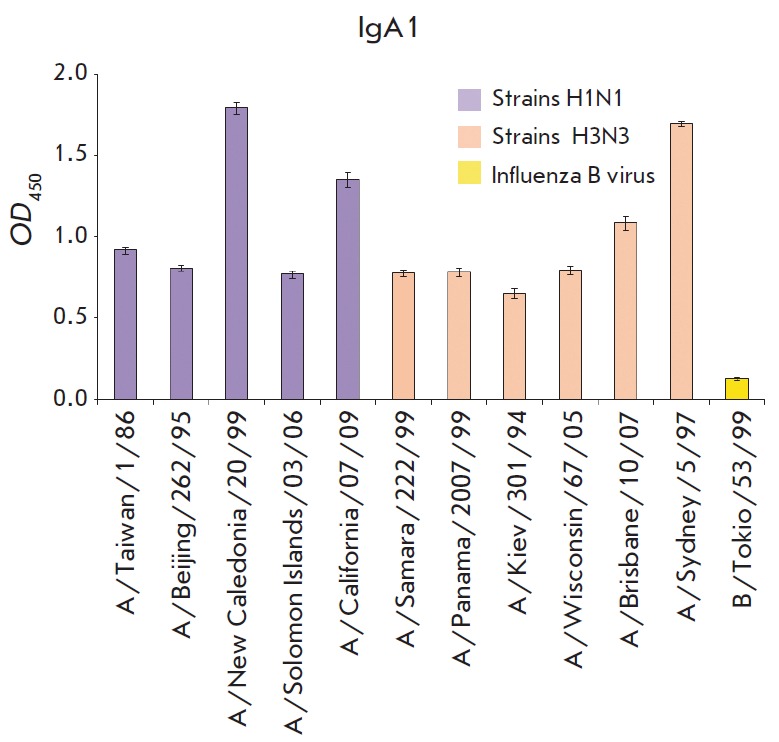
Indirect ELISA of the interaction of influenza A strains of H1N1 and H3N2 subtypes with recombinant FI6 antibodies of IgA1 isotype.

**Fig. 5:**
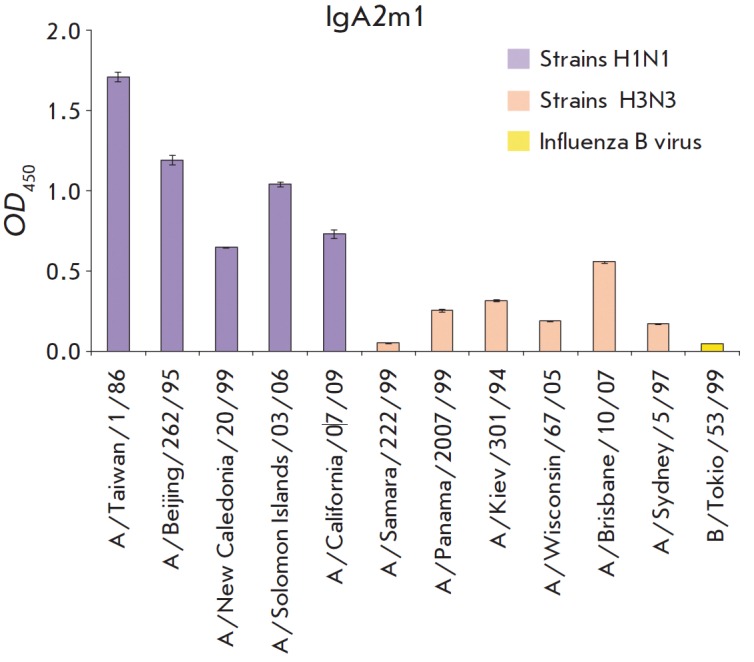
Indirect ELISA of the interaction of influenza A strains of H1N1 and H3N2 subtypes with recombinant FI6 antibodies of IgA2m1 isotype.

The immunochemical analysis (*Fig. 4* and *Fig. 5*) indicates that recombinant IgA1- and IgA2m1-isotype antibodies recognize the strains of both isotypes from different phylogenetic groups. The affinity of recombinant IgA1- and IgA2m1-isotype antibodies to some strains of the investigated subtypes is different. The greatest difference is observed for strains of the H3N2 subtype; the intensity of H3N2 interaction with the IgA2m1-isotope antibodies is much lower than that with IgA1-isotype antibodies.

Our data agree with the data [8] showing that FI6 antibodies recognize 16 subtypes of IAV and exert different binding strengths on different virus subtypes.

For the three obtained recombinant antibodies, the dissociation constants of the antigen–antibody complex were estimated for the A(H1N1)/Solomon Islands/03/06 influenza strain antigen (*Table 2*).

**Table 2:** Comparison of K_d_ values for FI6 recombinant antibodies of IgG and IgA isotypes

Antibody	K_d_, nM
IgG1	1.2–1.8
IgA1	0.7–1.5
IgA2m1	3.3–3.9

The *K*d values of the recombinant IgA1 and IgA2m1 antibodies differ considerably (4 times). Herewith, the *K*d value of IgA1 is even slightly lower than that of IgG1, which indicates a greater binding strength and is probably caused by the greater flexibility of the antigen-binding sites of the variable domains associated with the unique IgA1 hinge site structure. Overall, our study shows that production of the IgA isotype FI6 antibody does not deteriorate its antigen-binding properties. It is noted that retaining high degrees of antigen-binding and neutralizing properties when reformatting an antibody isotype is not an obvious result as indicated by the data in [22]. It was shown that the chimeric (mouse-human) antibody 9F4 of IgA1 isotype to H5N1 subtype hemagglutinin exerts a lower neutralizing activity compared to parental mouse antibody and the chimeric version of the IgG-isotype.

## CONCLUSION

Recombinant monomer antibodies of IgA1- and IgA2m1-isotypes on the basis of variable domains of the broad-spectrum antibody FI6 to influenza A virus hemagglutinin were obtained. These antibodies recognize 10 relic and current IAV strains in indirect ELISA and are characterized by a *K*_d_ value of the antigen–antibody complex no higher than 4 nM. The affinity of the studied antibody samples to the IAV strains of the H1N1 subtype is higher than the affinity to the H3N2 subtype strain. Our data show that production of antibody FI6 of monomer IgA form does not change its antigen-binding properties, which is an important prerequisite for the use of IgA-isotype antibodies for therapeutic purposes.

## Abbreviations

IAV: influenza A virus
MES: 2-(N-morpholino)ethanosulfonic acid
ELISA: enhanced light immunosorbent assay
Kd: dissociation constant
